# Natural Products with Toll-Like Receptor 4 Antagonist Activity

**DOI:** 10.1155/2018/2859135

**Published:** 2018-03-01

**Authors:** Monica Molteni, Annalisa Bosi, Carlo Rossetti

**Affiliations:** Dipartimento di Biotecnologie e Scienze della Vita, Università degli Studi dell'Insubria, Via Dunant, 3-21100 Varese, Italy

## Abstract

Toll-Like Receptors (TLRs) are the innate immunity receptors that play an activating role when interacting with molecules released by bacteria and viruses (PAMPs, pathogen-associated molecular patterns) or with molecules released by injured cells and tissues (DAMPs, danger-associated molecular patterns). TLR triggering leads to the induction of proinflammatory cytokines and chemokines, driving the activation of both innate and adaptive immunity. In particular, Toll-Like Receptor 4 (TLR4) has been described to be involved in the inflammatory processes observed in several pathologies (such as ischemia/reperfusion injury, neuropathic pain, neurodegenerative diseases, and cancer). Molecules obtained by natural sources have been discovered to exert an anti-inflammatory action by targeting TLR4 activation pathways. This review focuses on TLR4 antagonists obtained from bacteria, cyanobacteria, and plants.

## 1. Introduction

Toll-Like Receptor 4 (TLR4) belongs to the family of pattern recognition receptors (PRRs), conserved receptors of innate immunity, homologues of the* Drosophila* Toll protein, discovered to be important for the defense against microbial infections. TLRs are highly conserved from* Drosophila* to humans and share structural and functional similarities [1, 2]. These innate immune receptors recognize pathogen-associated molecular patterns (PAMPs) expressed by infectious agents and have a key role in directing the development of an effective immune response against pathogens [3]. Evolutionarily, the innate immune system is more ancient than the adaptive immune system. Differently from adaptive immunity, innate immune recognition is characterized by germ-line–encoded receptors; thus the specificity of each receptor is genetically predetermined. One of the advantages of innate immune receptors is that they had evolved by natural selection to recognize a few highly conserved structures shared by large groups of microorganisms (Table 1). For instance, all gram-negative bacteria have lipopolysaccharides (LPS); therefore, the lipopolysaccharide pattern recognition receptor of the host (e.g., TLR4) can detect the presence of virtually any gram-negative bacterial infection [4]. Indeed, it has been demonstrated that some TLRs, and particularly TLR4, respond to danger-associated molecular patterns (DAMPs) that are endogenous molecules of the host, released by injured tissue and dying cells [5] (Table 1). The stimulation of TLRs by the corresponding PAMP or DAMP initiates intracellular signaling cascades leading to the activation of transcription factors, such as AP-1, NF-*κ*B, and interferon regulatory factors (IRFs) [6]. Signaling by TLRs results in a variety of cellular responses including the production of proinflammatory cytokines, type I interferons (IFNs), and effector cytokines that direct both innate and adaptive immune responses [7].

Although TLR-mediated signaling has a leading role in both eradicating microbial infections and promoting tissue repair, the regulation must be tight. TLRs are implicated in a number of infectious and noninfectious diseases and immune disorders, as well as in cancer; they can either promote or inhibit disease progression [4, 8–11]. The importance of TLRs triggering in infectious diseases is evident, as they are the main receptors of innate immunity involved in sensing bacterial, fungal, and viral infections; indeed, it has been recently demonstrated that TLRs, and particularly TLR4, are involved in noninfectious diseases [reviewed in [4]]. TLR4 engagement by endogenous ligands has been demonstrated to directly contribute to the process of ischemia/reperfusion injury. Furthermore, in neuroinflammation, which is the common hallmark of several neurodegenerative and neurological diseases, TLR4 has been demonstrated to represent a critical amplifier of the proinflammatory response [4, 12]. In experimental models of systemic lupus erythematosus, TLR7 and TLR9 have been shown to play important roles in the production of pathogenic autoantibodies and/or in the development of clinical signs of autoimmunity [8]. TLRs are also expressed on tumor cells, where they may influence tumor growth and host immune responses [13]. It has been demonstrated that melanoma cells express TLR4, and this expression is particularly high in metastatic cells [9]. Triggering of TLR4 on tumor cells by LPS induces the release of several mediators that can favor tumor cell resistance to cytotoxic lymphocytes, reduces apoptosis, and increases invasiveness [9, 13]. TLR targeting could represent a means to regulate the immune response; however, therapeutic agents must be able to antagonize the harmful effects of TLR engagement, without affecting host defense functions. Several natural products targeting TLRs have been described; in this context TLR4 is the prototype, not only for its central role in several infectious and noninfectious inflammatory diseases both, but also because several products from natural sources targeting this receptor have been discovered with agonist or antagonist function. In this review, natural molecules with TLR4 antagonist activity will be described.

## 2. TLR4 Structure and Signaling

**Figure 1:**
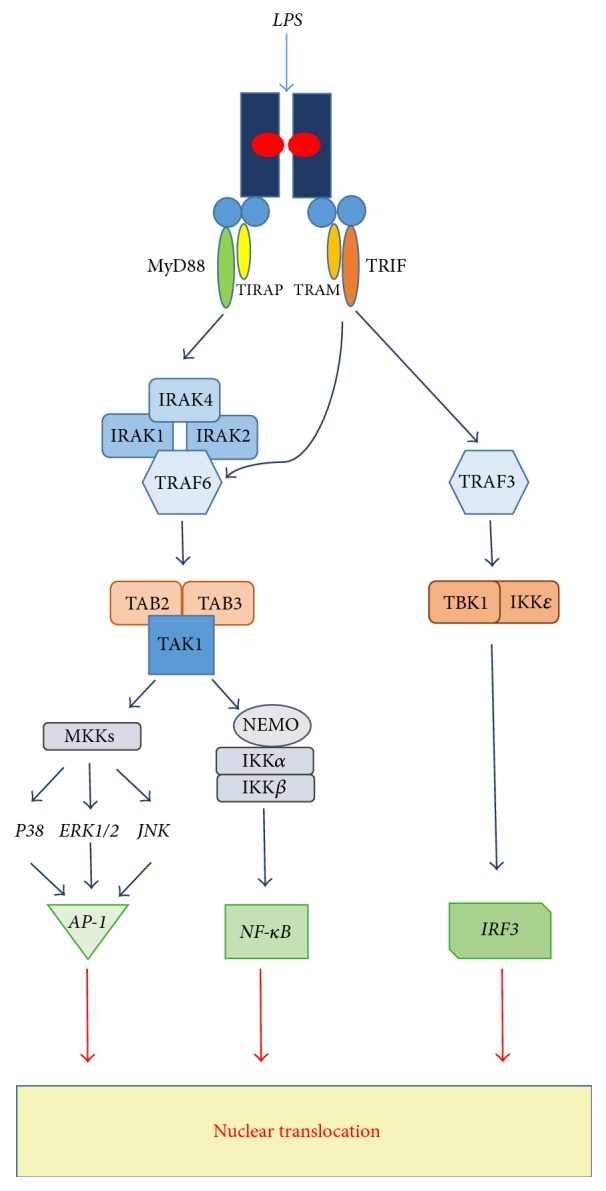
TLR4 intracellular signaling pathways. TLR4 signaling is induced by interaction with the specific ligand (e.g., LPS). In detail, LPS binding to MD-2 promotes dimerization of TLR4/MD-2 with the recruitment of intracellular adaptor proteins, MyD88 and TIRAP (MyD88-dependent pathway) or TRIF and TRAM (MyD88-independent pathway). In MyD88-dependent pathway, there is the recruitment and activation of IRAKs and TRAF6, inducing TAK1 activation. TAK1 coupling to the IKK complex and NEMO leads to IkB phosphorylation and nuclear translocation of NF-*κ*B. TAK1-dependent activation of MKKs promotes AP-1 transcription factor induction. In MyD88-independent pathway TRIF and TRAM adaptor proteins are involved in the activation of TRAF3 and, downstream, in the induction of TBK1 and IKK*ε*, needed for the activation of the transcription factor IRF3.

TLR4 is characterized by an extracellular domain composed of 608 residues and an intracellular domain of 187 residues. The intracellular domain is involved in the signaling cascade, consisting of at least two distinct pathways: MyD88-dependent pathway that leads to the production of proinflammatory cytokines and MyD88-independent pathway associated with the stimulation of type I IFNs [14]. MyD88-dependent pathway is common to all TLRs, except TLR3 [6]. TLR4 signaling responds to signals, such as LPS, by forming a complex using an extracellular leucine-rich repeat domain (LRR) and an intracellular toll/interleukin-1 receptor (TIR) domain. LPS induces a series of interactions with several accessory proteins, which form the TLR4 complex on the cell surface. LPS recognition is initiated by the binding of LPS to an LPS Binding Protein (LBP) [15]. The LPS-LBP complex transfers the LPS to CD14. CD14 is a glycosylphosphatidylinositol-anchored membrane protein that binds the LPS-LBP complex and facilitates the transfer of LPS to Myeloid Differentiation- (MD-) 2 protein. Crystallographic studies showed that MD-2 possesses a hydrophobic pocket that hosts the nonpolar portion of LPS (e.g., lipid A) and is associated with the extracellular domain of TLR4. LPS binding to MD-2 promotes the dimerization of TLR4/MD-2 [16–18]. The conformational changes of TLR4 induce the recruitment of intracellular adaptor proteins containing the TIR domain that is necessary for activating the downstream signaling pathway. Adaptor proteins include the TIR domain containing proteins, MyD88, TIRAP (TIR-associated protein), TRIF (TIR domain containing adaptor protein-inducing IFN-*β*), and TRAM (TRIF-related adaptor molecule) (Figure 1). The activation of MyD88 pathway involves the recruitment of IRAK1 and IRAK4. IRAK4 activates IRAK1 by phosphorylation. Both IRAK1 and IRAK4 leave the MyD88-TLR complex and associate temporarily with TRAF6. Recently, IRAK2 was shown to play a central role in TRAF6 ubiquitination [18]. Following ubiquitination, TRAF6 forms a complex with TAB2/TAB3/TAK1 inducing TAK1 activation [15]. TAK1 then couples to the IKK complex, which includes the scaffold protein NEMO [19], leading to the phosphorylation of I*κ*B and the subsequent nuclear localization of NF-*κ*B. TAK1 also induces MAP kinase- (MKK-) mediated activation of p38, JNK, and ERK1/2 that are involved in the activation of transcription factor AP-1 [14, 20]. Activation of NF-*κ*B and AP-1 triggers the production of proinflammatory cytokines such as TNF-*α*, IL-1, and IL-12. MyD88-independent pathway involves TRIF and TRAM adaptor proteins, the activation of TRAF3, and downstream induction of TBK1 and IKK*ε*, which are responsible for the recruitment and activation of the transcription factor, IFN regulatory factor 3 (IRF3). IRF3 is involved in the activation of type I IFN productions (e.g., IFN*α* and IFN*β*) [20, 21].

## 3. Natural Products Targeting TLR4 with Antagonist Activity

Natural TLR4 antagonists were mainly obtained from gram-negative bacteria and cyanobacteria or from plants. In bacteria and cyanobacteria, molecules with TLR4 antagonist activity were structurally LPS or Lipooligosaccharides (LOS); in plants, antagonists were low molecular weight molecules structurally unrelated with LPS. Natural TLR4 antagonists were shown to exert their action in the extracellular compartment, by blocking the formation of the TLR4/MD-2 complex and acting either on CD14 or on MD-2.

The molecules of natural origin with well-demonstrated TLR4 antagonist activity, currently reported in the literature, were

  LPS and lipid A from* Rhodobacter sphaeroides*,
  LOS from* Bartonella quintana*,
  LPS from* Oscillatoria Planktothrix FP1*,
  curcumin from* Curcuma longa*,
  sulforaphane and iberin from cruciferous vegetables,
  xanthohumol from hops and beer,
  celastrol from* Tripterygium wilfordii*.

Other molecules from plants and herbs of traditional Chinese medicine, such as berberine, atractylenolide I, and zhankuic acid A, have been described as TLR4 antagonist molecules; indeed the mechanism of action has been only hypothesized on the basis of docking analysis and has not been experimentally demonstrated, yet [reviewed [27]].

## 4. TLR4 Antagonists from Bacteria and Cyanobacteria

### 4.1. LPS and Lipid A from* Rhodobacter sphaeroides*

**Figure 2:**
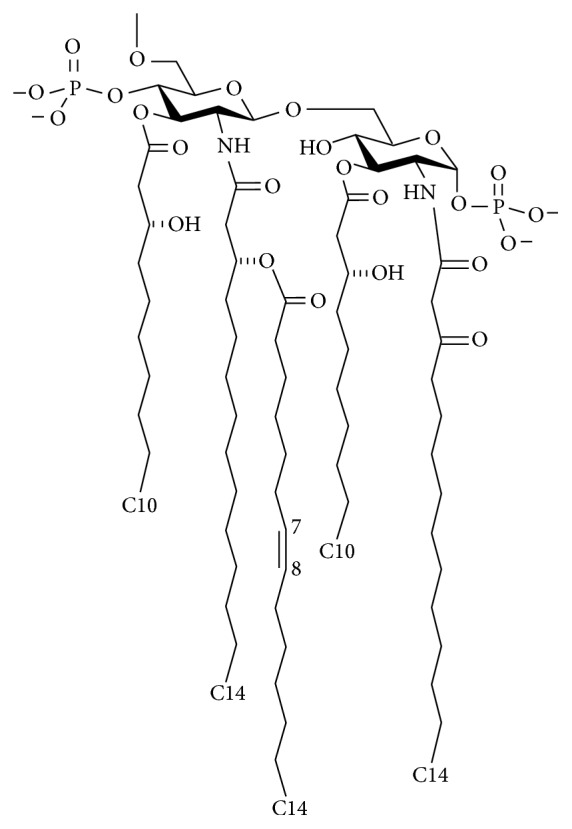
Structure of lipid A from* Rhodobacter sphaeroides*. Source: [22], under Creative Commons Attribution 4.0 International License.

LPS from* Rhodobacter sphaeroides* (RsLPS), a nonpathogenic photosynthetic gram-negative bacterium, was the first naturally occurring potent TLR4 antagonist to be discovered.* Rhodobacter* LPS was shown to be nontoxic [28] and to compete with toxic LPS for binding to LBP [29]. Further studies were done to elucidate the chemical structure of* Rhodobacter sphaeroides* lipid A (RsDPLA), the structural moiety obtained from RsLPS by mild acid hydrolysis, maintaining the biological activity. RsDPLA is a 1,4′-diphosphoryl penta-acyl lipid A. It consists of a D-glucosaminyl-*β*(1-6)-D-glucosamine backbone, carrying phosphate groups. The fatty acyl groups attached to the distal sugar unit of the lipid A are 3-hydroxydecanoic acid and Δ^7^-[(tetradecenoyl) oxy]-tetradecanoic acid. The fatty acyl groups attached to the reducing sugar unit are 3-hydroxydecanoic acid and oxotetradecanoic acid [30, 31] (Figure 2). RsDPLA biological activity was deeply studied both* in vitro* and* in vivo*, demonstrating a potent activity as antagonist of LPS in human and murine cells and preventing endotoxic shock in mice [32, 33]. It has been demonstrated that RsDPLA competes with LPS for binding to LBP and soluble CD14; indeed, recent computational studies showed that RsDPLA interacts with TLR4/MD-2 complex, acquiring antagonist configurations in humans and in mice and agonist-like configurations in horses and hamsters [34, 35]. RsDPLA has been used as a model to create synthetic antagonists (E5531, Eritoran) [36, 37] to be employed as drugs for the treatment of gram-negative sepsis. Eritoran that blocks LPS from binding to TLR4/MD-2 complex [38] was used in phase III clinical trial to evaluate whether it could be able to reduce sepsis-induced mortality. Unfortunately, results were disappointing [39], suggesting that sepsis is a very complex disease in which the early occurring “cytokine storm” is dependent only in part on TLR4 and is rapidly followed by profound immunosuppression that affects patients' survival [40].

### 4.2. LOS from* Bartonella quintana* (BqLOS)

**Figure 3:**
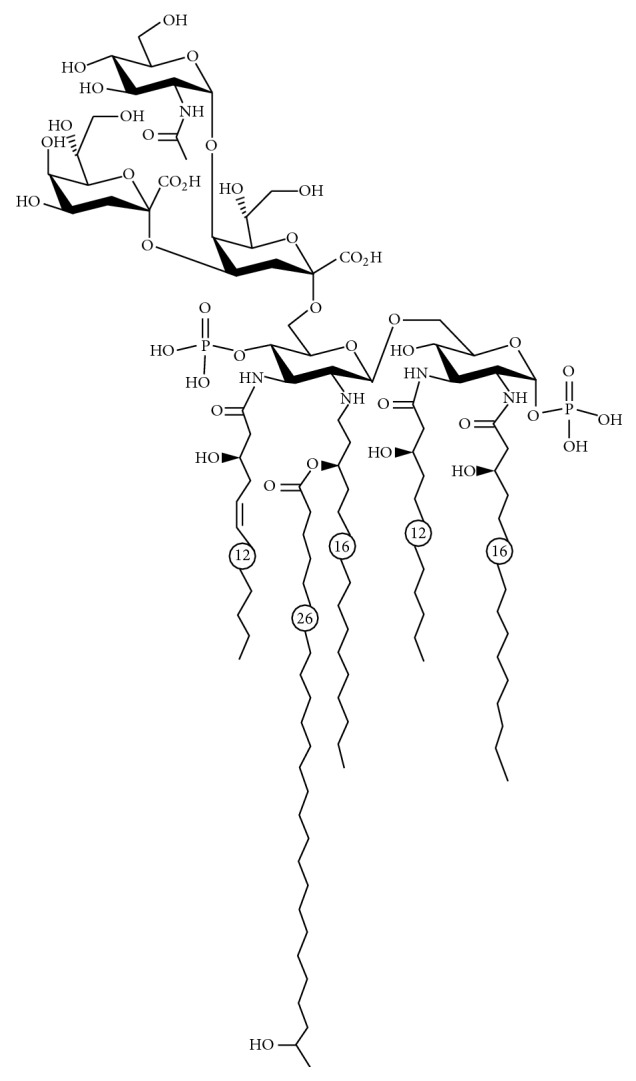
Proposed structure of LOS from* Bartonella quintana*. Source: [23], under Creative Common Attribution 4.0 International License.

LOS showing TLR4 antagonist activity from* Bartonella quintana* (BqLOS) was recently identified (Figure 3).* B. quintana* was initially described during World War I as the causative agent of trench fever.* B. quintana* is present in the bloodstream of patients during the febrile stage of trench fever; indeed, bacteremia can persist longer after the disappearance of all clinical signs [41, 42]. BqLOS is characterized by the presence of 1,4′-diphosphoryl penta-acyl lipid A. Fatty acid composition consists of 3-hydroxydodecanoic acid, 3-hydroxy-5-dodecenoic acid, and 3-hydroxyhexadecanoic acid; interestingly, also a long chained fatty acid (e.g., 3-hydroxyhexacosanoic acid) is present in the lipid A structure. It has been demonstrated that BqLOS specifically and rapidly binds TLR4, without transducing any intracellular signaling. Therefore, when added in culture together with* E. coli* LPS, it almost completely inhibited the production of proinflammatory mediators induced by* E. coli* LPS [23, 43]. Even though the mode of interaction with TLR4 has not been elucidated and further experiments are needed to clarify this point, it is more likely that the antagonist effect could be mediated by the interaction with MD-2. In experiments of endotoxemia in mice, using* E. coli* LPS and D-galactosamine to induce septic shock, a single injection of BqLOS 30 min before* E. coli* LPS plus D-galactosamine was shown to be protective [23]. Another study [44] showed reduced disease progression in collagen-induced arthritis in mice treated with BqLOS. The effect was mediated by a reduction of IL-1 expression in the joint and suggested the efficacy of TLR4 targeting in diseases in which endogenous TLR4 ligands are involved in the inflammatory process.

### 4.3. LPS from* Oscillatoria Planktothrix FP1* (Cyanobacterial Product, CyP)

**Figure 4:**
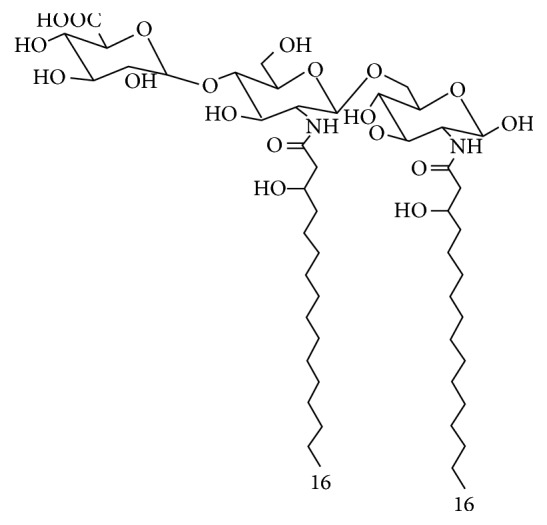
Proposed structure of lipid A from* Oscillatoria Planktothrix FP1.*

Cyanobacteria are microorganisms with a cell wall that structurally has intermediate characteristics between gram-negative and gram-positive bacteria. Similarly to gram-negative bacteria, cyanobacteria have LPS-like structures as main components of the outer membrane layer. A LPS-like structure (Cyanobacterial Product, CyP) with TLR4 antagonist activity was obtained from a cyanobacterium,* Oscillatoria Planktothrix FP1*. CyP structure is characterized by a rhamnose rich region in the O-antigen and by an inner oligosaccharidic core mainly composed of galacturonic acids [45]. Differently from gram-negative LPS agonists and antagonists, CyP lipid A is composed of an acylated disaccharide glucosamine backbone devoid of phosphate groups, linked to a galacturonic acid. Acylation pattern showed the presence of at least two 3 hydroxy-hexadecanoic acids (Figure 4) [45]. It has been demonstrated that CyP is highly efficient in antagonizing the effects of LPS through a specific interaction with MD-2, thus blocking LPS from binding to TLR4/MD-2 complex in the extracellular compartment [46, 47]. CyP inhibited both MyD88- and TRIF-dependent pathways activated by bacterial LPS, suppressing the whole LPS-induced gene transcription program in human monocyte-derived dendritic cells [46]. Differently from the other bacterial TLR4 antagonists, CyP was active in the inhibition of proinflammatory cytokines induced by LPS* in vitro* even when added several hours after LPS [46]. Furthermore, CyP is not species-specific since it was active in human, mouse, and porcine cells [46–48]. Results of* in vivo* studies showed that TLR4 antagonism by CyP could be effective in the treatment of noninfectious diseases, in which detrimental, TLR4-driven inflammatory processes induced by endogenous ligands play a pivotal role. Interesting results were observed in animal models of neurological and neurodegenerative diseases, such as in epilepsy, models of Amyotrophic Lateral Sclerosis, and Alzheimer's diseases [49–52]. In mice models of seizures, a TLR4-HMGB1 pathway having proconvulsant effects has been described [49]. TLR4 antagonism by CyP was effective in delaying seizure onset and in reducing recurrence in an acute model of seizure; the treatment with CyP in combination with a drug targeting IL1R1 (VX-765) after epilepsy onset in a chronic mice model prevented disease progression and drastically reduced chronic seizure recurrence [50].

## 5. TLR4 Antagonists from Plants

### 5.1. Curcumin from* Curcuma longa*

**Figure 5:**
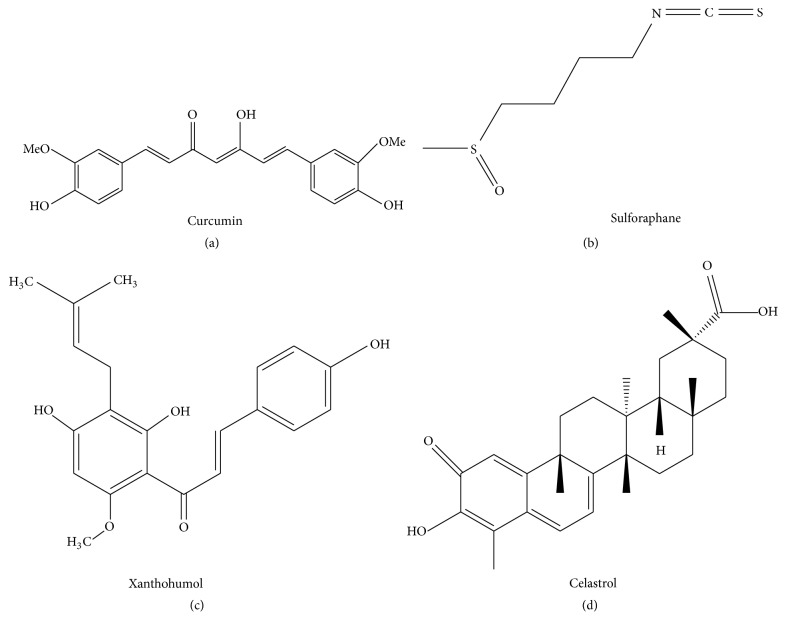
Structure of curcumin (a) and sulforaphane (b), xanthohumol (c), and celastrol (d). Sources: curcumin structure: [24], under Creative Commons Noncommercial No Derivative Works (CC-BY-NC-ND) Attribution License. Sulforaphane structure: [25], under Creative Commons Attribution 4.0 International License. Xanthohumol structure: [26], under Creative Commons Attribution License (CC-BY). Celastrol structure: [27], under Creative Commons License (CC-BY).

Turmeric has been consumed by humans as a curry spice for centuries and is a well-known phytochemical used in traditional Indian and Chinese medicine for its anti-inflammatory action [53]. Turmeric contains, as a major compound, curcumin [(1E,6E)-1,7-bis-(4-hydroxy-3-methoxyphenyl)-1,6-heptadiene-3,5-dione], a polyphenolic molecule obtained by rhizomes of the plant* Curcuma longa* (Figure 5(a)). It has been demonstrated that curcumin binds noncovalently to MD-2, thus competing with LPS for TLR4/MD-2 complex [54]. Curcumin competition with LPS was responsible for inhibition of both MyD88-dependent and TRIF-dependent pathways [55]. It has been recently demonstrated that curcumin can modulate macrophage polarization through TLR4-mediated signaling inhibition [56].* In vivo*, in experimental model of traumatic brain injury, curcumin injection after injury significantly reduced microglial activation and brain injury via TLR4 pathways inhibition [57]. Curcumin was also shown to improve TNBS-induced colitis in mice [58]. Based on the results indicating a possible positive effect of curcumin on chronic colon inflammatory diseases, a new formulation of curcumin complexed with a polymer (Ora-curcumin) has been recently developed. The polymeric complex has been demonstrated to be have a better water solubility than curcumin alone and maintained a TLR4-antagonist activity* in vitro* [59]. Indeed, a recent paper by Nelson and colleagues [24] expressed great criticism about the possibility of considering curcumin as a drug lead for several reasons, among them chemical instability, low water solubility, lack of potent and selective target activity, and poor pharmacokinetic properties. Furthermore, all the double blinded, placebo controlled clinical trials employing curcumin failed to demonstrate any effect [reviewed in [24]], thus casting a dark shadow on the results published in the literature demonstrating curcumin activity.

### 5.2. Sulforaphane (SFN) and Iberin from Cruciferous Vegetables

Sulforaphane (SFN) [1-isothiocyanato-4-(methylsulfinyl)butane] is a natural occurring compound found in cruciferous vegetables. The chemical structure is reported in Figure 5(b). It has been demonstrated that SFN exerts anti-inflammatory effects by reducing the production of proinflammatory mediators, such as TNF-*α*, inducible NO synthase, cyclooxygenase-2, and HMGB1 secretion induced by LPS in macrophages [60, 61]. Experiments to elucidate the mechanism of action evidenced that SFN acts as an anti-inflammatory molecule at least in part by suppressing TLR4 oligomerization [60–62]. A recent paper by Koo and colleagues [63] showed that SFN blocks LPS interaction with TLR4/MD-2 complex by preferentially binding with MD-2. Indeed, similar to curcumin, all the biological effects ascribed to SFN were only in part dependent on the antagonism at the level of TLR4/MD-2 complex, thus showing low target selectivity [60–62].

An analogue of SFN, iberin [3-methylsulfinylpropyl isothiocyanate], showed a similar inhibitory effect on TLRs dimerization. The mechanism of action was not selective, since the disruption of TLR dimerization occurred on both TLR4 and TLR2, by covalent binding.* In vivo*, oral preadministration of iberin 1 h before LPS challenge inhibited LPS-induced proinflammatory cytokine production [64].

### 5.3. Xanthohumol from Hops and Beer

Xanthohumol (XN) is a chalcone-type flavonoid of* Humulus lupulus* (Figure 5(c)) showing anti-inflammatory effects [65, 66]. The anti-inflammatory action was observed on different stimulation pathways, such as those mediated by LPS and IFN-*γ*, in murine and human macrophages [65, 66]. Among the different mechanisms of action ascribed to XN [26, 67], it has been hypothesized that XN can also antagonize TLR4 activation by binding to MD-2, directly [66]. Recently, Fu and colleagues [68] confirmed this hypothesis by experimental methods. Using surface plasmon resonance they showed that XN binds to MD-2 in a dose-dependent manner with a relatively high affinity. Moreover, in a competitive biotin-streptavidin-based ELISA test, XN significantly reduced the binding of biotin-LPS to recombinant human MD-2, thus suggesting that XN has the same binding site as LPS [68]. These results indicate that XN anti-inflammatory effects are mediated, at least in part by an antagonist activity on TLR4/MD-2 complex.

### 5.4. Celastrol from* Tripterygium wilfordii*

Celastrol is a pentacyclic triterpenoid (Figure 5(d)) isolated from the root extract of* Tripterygium wilfordii *Hook F., used in traditional Chinese medicine to treat various inflammatory and autoimmune diseases, such as rheumatoid arthritis [69–71]. Celastrol has been demonstrated to form covalent Michael adducts with cysteine residues [69]. Based on this nonselective activity, among the different targets of celastrol, there is also MD-2 [27, 71, 72].

## 6. Conclusions

Several natural products from microorganisms and from plants targeting TLR4 have been discovered. Nevertheless, only molecules obtained from bacteria and cyanobacteria have been demonstrated to possess the necessary stability and target selectivity to be considered as models for the development of new drug candidates. Plant-derived low molecular weight compounds interacting with TLR4 can be hardly considered as models for the development of new therapeutics, since these molecules showed multiple cellular targets and their anti-inflammatory effects can be only partially ascribed to TLR4 antagonism.

Although the first attempts to obtain new drug lead by chemical synthesis, using natural antagonists from bacteria as models, did not reach positive results in clinical trials, the results obtained with these synthetic analogues helped to understand in more detail the mechanisms of interaction among the TLR4/MD-2 complex and different ligands. Major efforts are needed to obtain and to study new biologics targeting TLR4, since this could be of great value to have new modulators able to control the excessive inflammatory response observed in several pathological conditions.

## Figures and Tables

**Table 1:** Ligands and immune cells expressing Toll-Like Receptors. Mo, monocytes; MΦ, macrophages, DC, dendritic cells; MC, mast cells; B, B lymphocytes; T, T lymphocytes; NK, NK cells; HSPs, heat shock proteins; HMGB1, high mobility group box 1; mRNA, messenger RNA; ssRNA, single-stranded RNA.

TLR	Subcellular	Immune cell	PAMPs	DAMPs
	localization	expression		
TLR1/TLR2	Plasma membrane	Mo, MΦ, DC, B	Triacylated lipoproteins Peptidoglycan Lipopolysaccharide	



TLR2/TLR6	Plasma membrane	Mo, MΦ, MC, B	Diacylated lipopeptides Lipoteichoic acid Zymosan	HSPs HMGB1 Versican Hyaluronan

TLR3	Endosome	B, T, NK, DC	Double strand RNA	mRNA

TLR4	Plasma membrane/endosome	Mo, MΦ, DC, MC	Lipopolysaccharide F protein of syncytial virus Mannuronic acid polymers Teichuronic acid Flavolipin Mannan NS1 protein of dengue virus	HSPs HMGB1 Hyaluronan Biglycan Fibronectin Heparan sulphate Tenascin C

TLR5	Plasma membrane	Mo, MΦ, DC	Flagellin	

TLR7	Endosome	Mo, MΦ, DC, B	ssRNA	ssRNA

TLR8	Endosome	Mo, MΦ, DC, MC	ssRNA	ssRNA

TLR9	Endosome	Mo, MΦ, DC, B, T	CpG DNA	Chromatin IgG complex

TLR10/TLR2 TLR10	Plasma membrane/ endosome	Mo, DC, B	Gram-positive molecules Influenza virus molecules	

